# Spatiotemporal Patterns of Diarrhea Incidence in Ghana and the Impact of Meteorological and Socio-Demographic Factors

**DOI:** 10.3389/fepid.2022.871232

**Published:** 2022-04-08

**Authors:** Ernest O. Asare, Joshua L. Warren, Virginia E. Pitzer

**Affiliations:** ^1^Department of Epidemiology of Microbial Diseases, Yale School of Public Health, Yale University, New Haven, CT, United States; ^2^Public Health Modeling Unit, Yale School of Public Health, Yale University, New Haven, CT, United States; ^3^Department of Biostatistics, Yale School of Public Health, Yale University, New Haven, CT, United States

**Keywords:** climate and diarrhea, socio-demographic factors and diarrhea, weather and diarrhea, diarrhea in Ghana, diarrhea patterns, diarrhea modeling, diarrhea and floods

## Abstract

**Background:**

Diarrhea remains a significant public health problem and poses a considerable financial burden on Ghana's health insurance scheme. In order to prioritize district-level hotspots of diarrhea incidence for effective targeted interventions, it is important to understand the potential drivers of spatiotemporal patterns of diarrhea. We aimed to identify the spatiotemporal heterogeneity of diarrhea incidence in Ghana and explore how meteorological and socio-demographic factors influence the patterns.

**Methods:**

We used monthly district-level clinically diagnosed diarrhea data between 2012 and 2018 obtained from the Center for Health Information and Management of the Ghana Health Services. We utilized a hierarchical Bayesian spatiotemporal modeling framework to evaluate potential associations between district-level monthly diarrhea incidence and meteorological variables (mean temperature, diurnal temperature range, surface water presence) and socio-demographic factors (population density, Gini index, District League Table score) in Ghana. In addition, we investigated whether these associations were consistent across the four agro-ecological zones.

**Results:**

There was considerable spatial heterogeneity in diarrhea patterns across the districts, with clusters of high diarrhea risk areas mostly found in the transition and savannah zones. The average monthly temporal patterns of diarrhea revealed a weak biannual seasonality with major and minor peaks in June and October, respectively, coinciding with the major and minor rainy seasons. We found a significant association between both meteorological and socio-demographic factors and diarrhea risk, but the strength and direction of associations differed across the four agro-ecological zones. Surface water presence demonstrated consistently positive, while diurnal temperature range and population density demonstrated consistently negative associations with diarrhea both overall and across the agro-ecological zones.

**Conclusions:**

Although overall diarrhea incidence is declining in Ghana, our results revealed high-risk districts that could benefit from district-specific tailored intervention strategies to improve control efforts. Ghana health sector policy-makers can use these results to assess the effectiveness of ongoing interventions at the district level and prioritize resource allocation for diarrhea control.

## Introduction

An increase in diarrhea control strategies, particularly improvements in water, sanitation, and hygiene (WASH) infrastructure and improvements in access, treatment, and quality of health services, has contributed to significant reductions in diarrhea morbidity and mortality over the past four decades (1). For instance, diarrhea has dropped from being the second leading cause of global disease burden (as measured by disability-adjusted life-years) in 1990 to the ninth leading cause in 2020 (2). Nevertheless, diarrhea remains one of the most common preventable causes of morbidity and mortality, responsible for 1.6 and over 0.5 million deaths among all age groups and children under 5, respectively (3). This highlights the need to sustain current control interventions and identify novel effective strategies, particularly in low-income settings where diarrhea burden remains highest.

Diarrhea incidence is highly heterogeneous, both within and between countries, exhibiting substantial spatial and temporal variability (4–7). These variations are driven largely by meteorological factors (8, 9), socio-economic status and WASH infrastructure (10–15), and access and quality of health services (16). The interaction of these factors can create spatial and temporal hotspots of diarrhea incidence. For example, Bandyopadhyay et al. (5) found a decrease in monthly rainfall and increase in temperature during the dry season was able to delineate hotspots of diarrhea prevalence in children under the age of 3 years in sub-Saharan Africa. In Anhui Province of China, Hao et al. (17) found a strong association between socio-economic status and spatial clustering of diarrhea incidence. These studies demonstrate that the combination of meteorological and socio-demographic factors create different suitable pathways for diarrhea transmission, which are likely to be setting-specific. Understanding and identifying hotspots and the potential driving factors is critical to develop setting-specific control strategies to reduce diarrhea burden.

In Ghana, similar to other low-income countries, diarrhea ranks among the top ten causes of morbidity and mortality. In 2015, the estimated number of diarrhea-associated deaths among all ages and children under 5 years were approximately 3,800 and 1,700, respectively (18). In addition to the health burden of diarrhea, there is also a significant economic burden on both individuals and the country (19). In northern Ghana, Aikins et al. (20) estimated the treatment cost per outpatient and inpatient diarrhea to be US$3.86–4.35 and US$65.14–133.86 (based on 2003 and 2004 US dollars), respectively depending upon treatment regime, which is high for the low-income population. The main treatment for both inpatients and outpatients with diarrhea includes administration of oral rehydration solutions and antibiotics (20). Therefore, reducing the diarrhea burden in Ghana should have both health and economic benefits.

A number of models have been developed to predict spatiotemporal patterns of diarrhea incidence using a combination of environmental and socio-economic factors (6, 9, 21, 22). While these models tend to provide reliable estimates of diarrhea patterns, they are often carried out over large spatial scales, particularly at country and state levels. Thus, it is important to accurately model the spatiotemporal diarrhea patterns at finer spatial scales to provide useful information to evaluate current control programs at the local level.

To address this, we used a hierarchical Bayesian spatiotemporal modeling framework to quantify associations between meteorological and socio-demographic factors and district-level diarrhea incidence in Ghana between 2012 and 2018. We also investigated variations in the impact of these factors on diarrhea across the four agro-ecological zones in the country. The results can help policy-makers to identify high-risk diarrhea districts for which improved interventions are needed.

## Methods and Data

### Diarrhea Data and Study Area Description

Monthly district-level diarrhea morbidity records were obtained from the Center for Health Information and Management (CHIM) of the Ghana Health Services (GHS) for 2012 to 2018. Diarrhea was clinically diagnosed, and methods of detection and reporting were consistent across districts. Currently, Ghana consists of 16 regions and 260 districts. However, our data consist of 216 districts between 2012 and 2014 and 260 districts beginning in 2015. In order to use all the available data, we merged the diarrhea data for districts that were split from the original 216 in 2014. Annual district-level population estimates were obtained from the Ghana Statistical Service (GSS). We calculated the monthly crude incidence of diarrhea per 10,000 persons for each district by dividing the monthly diarrhea cases by the district population.

### Meteorological Data

We used daily rainfall estimates from the Climate Hazards group Infrared Precipitation with Stations (CHIRPS) with a spatial resolution of 0.05° X 0.05° (23). Monthly mean (Tmean), minimum (Tmin), and maximum (Tmax) 2-meter air temperature data were obtained from the European Center for Medium-Range Weather Forecasts (ECMWF) fifth-generation reanalysis ERA5 (24), which has a spatial resolution of 0.25° X 0.25°. Both datasets were extracted over Ghana for the study period 2012 to 2018. The daily Tmax and Tmin were used to calculate the diurnal temperature range (dtr = Tmax-Tmin), which is inversely associated with humidity. The daily dtr was averaged for each month. Tmean and dtr were included as explanatory variables because they have been shown to influence the survival of diarrheal pathogens (25, 26).

The rainfall data were used to run a surface hydrology model (used as a proxy for persistent flooding) developed by Asare et al. (27, 28) that predicts fractional surface water presence (wpre) in each grid cell at each time step. The remaining model inputs are derived; the model equation is given by:


(1) $$\begin{array}{l} \frac{d\omega_{\text{pond}}}{dt}=\frac{2}{\rho h_{\text{ref}}}{\left(\frac{\omega_{\text{ref}}}{\omega_{pond}}\right)}^{\frac{\text{ρ}s}{2}}(\left(Q\left(\omega_{\max}-\omega_{\text{pond}}\right)+P\omega_{pond}\right)\;\\ \;\left(1-f\right)-\omega_{\text{pond}}\left(E+fI_{\max}\right)) \end{array}$$

where ω_pond_ is the daily fractional water coverage in a grid cell, ρs is the shape factor, ω_max_ is the maximum water coverage, *h*_ref_ is the reference water depth, ω_*ref*_ is the reference fractional water coverage, *I*_max_ is the maximum infiltration which is controlled by the scaling factor ($f=\frac{\omega_{\text{pond}}}{\omega_{\max}}$), *P* is the rainfall, *E* is the evaporation, and *Q* is estimated based on the soil conservation service curve number (SCS-CN) method (29). The fixed model parameters are provided in Table 1. The daily model output is aggregated to monthly temporal resolutions. We included wpre in the model because it plays an important role in the concentration, transport and distribution of diarrheal pathogens (31–33).

**Table 1:** Set of fixed parameters used in the surface water presence model (Equation 1).

**Parameter**	**Value**	**Reference**
ω_max_	0.1	(30)
ω_ref_	0.005	(30)
h_ref_ (mm)	250	(30)
ρs	1.5	(30)
CN	90	(29)
E (mm)	5	(30)
I_max_ (mm)	250	(30)

As a preliminary analysis, we examined the Pearson's correlations between the meteorological factors and monthly district-level diarrhea incidence.

### Socio-Demographic Data

We used the Gini index (34) as a proxy for socio-economic inequality. The Gini index ranges from 0 to 100%, with the highest values corresponding to high levels of income inequality. The district-level Gini index data were obtained from the 2015 report on poverty mapping in Ghana (35), which was derived based on the 2010 Population and Housing Census Data. We assumed constant Gini index over the study period; therefore, this variable only captures potential spatial variation in diarrhea incidence.

We used the Ghana District League Table (DLT) score, an index that represents the overall level of development across the districts (36). The DLT index is generated by aggregating seven key sector indicators (health, water, education, sanitation, security, governance, and child protection) into a single score ranging from 0 to 100%. Since the DLT index is only available from 2014, we used the 2014 index for 2012 and 2013.

The population density data were obtained from the WorldPop database (https://www.worldpop.org/). This gridded dataset has a spatial resolution of 1 km. The district-level population density data were estimated by averaging all the grid cell values within each district polygon.

### Statistical Analysis

We utilized a hierarchical Bayesian spatiotemporal modeling framework to quantify how meteorological and socio-demographic factors influence the spatiotemporal heterogeneity of diarrhea incidence in Ghana. The number of diarrhea cases in district *k* (*k* = 1, 2, …, 216) and month *t* (*t* = 1, 2, …, 84), *Y*_*kt*_, were assumed to follow a Poisson distribution with mean λ_*kt*_ such that


(2) $$\begin{array}{l} Y_{kt}\;\sim \text{ Poisson}\left({\text{λ}}_{kt}\right),\;\\ \ln\left({\text{λ}}_{kt}\right)=\ln\left(E_{kt}\right)+\;\rho_{kt} \end{array}$$

where ρ_*kt*_ and *E*_*kt*_ represent the log relative risk (RR) and expected number of cases in district *k* in month *t*, respectively. The expected number of cases was calculated as the monthly mean national diarrhea incidence rate multiplied by the population at risk in each district for each month.

The log relative risk, ρ_*kt*_, was modeled as a function of covariates and random effects, such that


(3) $$\begin{array}{l} \rho_{kt}=\beta_{0}+\beta_{1}\left({\text{wpre}}_{kt}\right)+\beta_{2}\left({\text{dtr}}_{kt}\right)+\beta_{3}\left({\text{Tmean}}_{kt}\right)\;\\ \;+\beta_{4}\left({\text{popden}}_{kt}\right)+\beta_{5}\left({\text{Gini}}_{k}\right)+\beta_{6}\left({\text{DLT}}_{kt}\right)+\phi_{k} \end{array}$$

where β_0_ is the intercept; β_1_, β_2_, β_3_, β_4_, β_5_, β_6_ are the regression coefficients for the explanatory variables, including the surface water presence (wpre), diurnal temperature range (dtr), mean temperature (Tmean), population density (popden), Gini index (Gini), and District League Table (DLT) score, respectively; and ϕ_*k*_ is the spatially-correlated random effect parameter from the *k*th district. We considered linear terms for all covariates without interactions because our focus was on the interpretability of the main effects. We used Pearson's correlation test to identify possible collinearity amongst the explanatory variables. The association between each of the covariates and diarrhea incidence was quantified in terms of RR for a one standard deviation increase in a covariate. The RR for the *i*th covariate (i.e., *RR*_*i*_) is calculated as exp (β_*i*_).

We modeled the spatially correlated random effects using the Leroux version of the conditional autoregressive model (37), which is best understood conditionally such that


(4) $$\phi_{k}|\phi_{-k}\sim N\left(\frac{\rho \sum_{i=1}^{216}w_{ki}\phi_{i}}{\rho \sum_{i=0}^{216}w_{ki}+1-\rho },\frac{\tau^{2}}{\rho \sum_{i=1}^{216}w_{ki}+1-\rho }\right)$$

where ϕ_−*k*_ is the complete vector of spatial random effect parameters with ϕ_*k*_ removed; *w*_*ki*_ = 1 if districts *k* and *i* share a common border and ω_*ki*_ = 0 otherwise (*w*_*kk*_ = 0 for all *k* by definition); ρ∈(0, 1) controls the strength of spatial correlation in the parameters with values near 1 indicating strong correlation and values near 0 suggesting independence; and τ^2^ is the variance of the random effects. This model suggests that a priori the random effect parameter from district *k* is normally distributed with the mean equal to a weighted average of its neighbors' values and variance defined as a function of the total number of neighbors it has.

### Prior Distributions

We used weakly informative prior distributions for each of the unknown model parameters to allow the data to drive the inference. The priors are specified as follows: β = (β_1_-β_6_) ~N (0,100^2^), σ^2^ ~ InverseGamma (1.00,0.01), τ^2^ ~ InverseGamma (1.00,0.01), and ρ ~ uniform (0, 1).

### Model Fitting

Model parameters were estimated using a Markov chain Monte Carlo (MCMC) sampling algorithm where we used three independent chains. Variables were standardized prior to model fitting for computational stability. We ran each chain for 320,000 iterations and discarded the first 220,000 as burn-in prior to convergence of the model. The remaining samples were thinned by a factor of 10, yielding 10,000 nearly independent posterior samples per chain for post-processing. Convergence was assessed by Geweke z-score diagnostics (38) for the model parameters. The models were fitted to both the whole district-level data and subsets of the data (the four agro-ecological zone: coastal, forest, transition and savannah zones) using the multi-level function S.CARmultilevel in the CAR-Bayes package in R (39).

## Results

**Figure 1:**
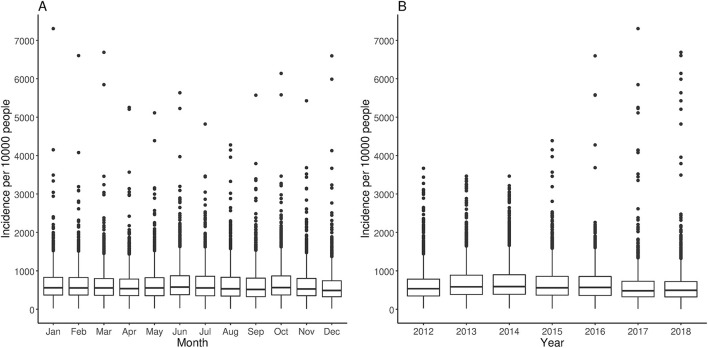
Average trends of diarrhea incidence across all districts between 2012 and 2018. **(A)** Monthly and **(B)** annual trends.

Between 2012 and 2018, the mean monthly diarrhea incidence was 643 (range: 4–7304) per 10,000 people (Table 2). The overall average district-level diarrhea incidence between 2012 and 2018 ranged from 80 (Akwapem South) to 2,978 (Bia West) per 10,000 people. The summary descriptive statistics of the variables for the agro-ecological zones are provided in Supplementary Table S1. The average monthly temporal pattern revealed a biannual incidence of diarrhea with slight peaks in June and October (Figure 1A). There was no substantial change in the mean annual trend in the overall diarrhea incidence (Figure 1B).

**Table 2:** Summary statistics of raw diarrhea incidence and potential explanatory variables.

**Variable**	**Mean (SD)**	**Range**	**Median (Quartiles)**
Diarrhea (per 10,000)	643.4 (449.2)	4.0, 7304.0	542.0 (352.0, 819.2)
dtr (°C)	0.547 (0.301)	0.000, 1.900	0.507 (0.321, 0.738)
Tmean (°C)	27.21 (1.796)	23.02, 34.30	27.01 (25.95, 28.13)
wpre	0.006 (0.005)	0.000, 0.023	0.006 (0.002, 0.009)
Gini index (%)	38.94 (6.118)	27.2, 64.00	37.40 (34.67, 42.27)
DLT score (%)	57.25 (11.40)	0.00, 100.00	58.68 (52.15, 64.97)
Population density (people/km^2^)	504.39 (1609.9)	8.7, 14118.8	120.5 (68.0, 265.0)

*wpre, surface water presence; dtr, diurnal temperature range; Tmean, mean temperature; DLT score, District League Table score*.

The Gini index varied between 27% (Upper Manya) and 64% (Sunyani Municipal), with a strong clustering along the western border of the country (Table 2 and Supplementary Figure S1A). The population density varied widely across the districts, ranging from 9 (North Gonja) to 14,119 (Accra Metropolitan Assembly) people per km^2^ (Table 2 and Supplementary Figure S1B). The DLT score varied between 0% (Asokore Mampong) and 100% (Asante Akim North) (Table 2 and Supplementary Figure S1C), with a substantial concentration of poorly developed districts mostly found in the eastern part of the country stretching between savannah and forest agro-ecological zones.

The district-level correlation coefficients between monthly diarrhea incidence and environmental factors ranged from −0.52 to 0.48 for Tmean (Supplementary Figure S2A), −0.51 to 0.37 for dtr (Supplementary Figure S2B), and −0.33 to 0.44 for wpre (Supplementary Figure S2C). Tmean and dtr were mostly positively correlated with diarrhea in the southern part of the country, while positive correlations with wpre were predominantly found in the northern part of Ghana. The Pearson's correlation matrix did not reveal high correlations between the covariates (|*r*| < 0.7) (Supplementary Table S2), suggesting the absence of collinearity.

For the spatiotemporal model, the Gini index, DLT score, and surface water presence were positively associated with diarrhea incidence, while mean temperature, diurnal temperature range, and population density showed negative associations with diarrhea incidence (Table 3). Population density had the most significant impact on diarrhea; a standard deviation (SD) decrease in population density was associated with a 50% (1/0.667) increase in diarrhea incidence. This was followed by the Gini index, for which a 1 SD increase was associated with a 16% increase in diarrhea incidence. The DLT score had the least significant impact on diarrhea, with 1 SD increase in DLT score associated with a 0.8% increase in diarrhea. The meteorological variable that showed the strongest association with diarrhea was diurnal temperature range, for which a 1 SD decrease was associated with a 2% increase in diarrhea incidence, whereas the effect of surface water presence and mean temperature were similar but opposite in direction (a 1.4% and 1.3% increase in diarrhea incidence for a 1 SD increase or decrease in wpre and Tmean, respectively). The Geweke z-score diagnostics are listed in Supplementary Table S3, with no obvious signs of non-convergence observed.

**Table 3:** Relative risk of diarrhea associated with meteorological and socio-demographic factors for the full spatiotemporal model.

**Variable**	**RR**	**2.5%**	**97.5%**
wpre	1.014	1.013	1.014
dtr	0.980	0.978	0.982
Tmean	0.987	0.986	0.988
Population density	0.667	0.659	0.673
Gini index	1.155	1.045	1.294
DLT score	1.008	1.007	1.009

*The posterior median and 95% quantile-based credible intervals are given for each covariate on the relative risk scale, with a one standard deviation change in covariate value interpretation (see Table 2). wpre, surface water presence; dtr, diurnal temperature range; Tmean, mean temperature; DLT score, District League Table score*.

The socio-demographic and meteorological factors were significant risk factors for diarrhea across the four agro-ecological zones (Table 4). However, there were differences in the strength and direction of associations across the different zones. Population density showed the greatest difference in relative risk across the agro-ecological zones; 165% (1/0.377) for the coastal zone and 4% (1/0.961) for the savannah zone. All the socio-demographic factors showed significant negative associations with diarrhea in the coastal zone. Surface water presence, diurnal temperature range and population density exhibited consistent but opposite directions of association with diarrhea incidence across the agro-ecological zones; surface water presence was positively associated with diarrhea incidence, while diurnal temperature range and population density were negatively associated (Table 4). There was a positive association between diarrhea incidence and mean temperature, except in the savannah zone. Positive associations between diarrhea incidence and DLT score were observed in the forest and savannah zones, whereas negative associations were found in the coastal and transition zones.

**Table 4:** Relative risk of diarrhea associated with meteorological and socio-demographic factors for the models fitted to each agro-ecological zone.

**Variable**	**Coastal**	**Forest**	**Transition**	**Savannah**
	**RR (95% CrI)**	**RR (95% CrI)**	**RR (95% CrI)**	**RR (95% CrI)**
wpre	1.021 (1.019, 1.023)	1.006 (1.004, 1.008)	1.016 (1.014, 1.017)	1.012 (1.010, 1.015)
dtr	0.973 (0.967, 0.982)	0.994 (0.991, 0.997)	0.960 (0.957, 0.963)	0.966 (0.963, 0.969)
Tmean	1.005 (1.002, 1.007)	1.009 (1.008, 1.011)	1.049 (1.047, 1.051)	0.941 (0.940, 0.943)
Population density	0.377 (0.366, 0.390)	0.868 (0.852, 0.884)	0.958 (0.946, 0.971)	0.961 (0.938, 0.983)
Gini index	0.884 (0.820, 0.934)	1.138 (1.011, 1.186)	1.203 (1.072, 1.324)	1.230 (1.182, 1.446)
DLT score	0.967 (0.965, 0.970)	1.027 (1.025, 1.029)	0.995 (0.993, 0.997)	1.017 (1.015, 1.019)

*The posterior median and 95% quantile-based credible intervals (95% Crl) are shown for each covariate for the four agro-ecological zones on the relative risk scale, with a one standard deviation change in covariate value interpretation. wpre, surface water presence; dtr, diurnal temperature range; Tmean, mean temperature; DLT score, District League Table score*.

**Figure 2:**
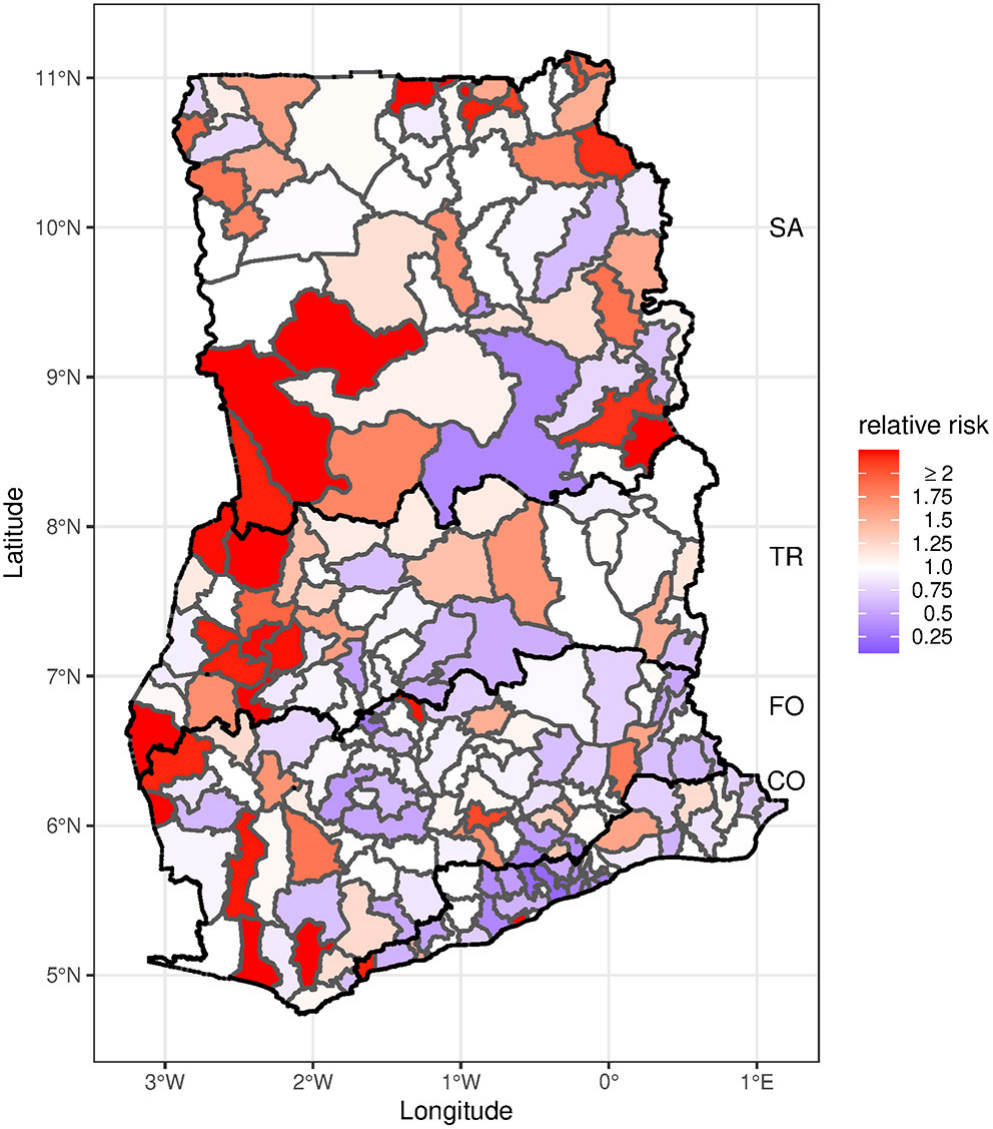
Map of posterior median estimates of the spatial random effects. Districts with lower than expected risk (RR <1) are in blue, while those with excess risk (RR >1) are in red. The black bold line indicates agro-ecological zone boundaries. CO, Coastal zone; FO, Forest zone; TR, Transition zone; SA, Savannah zone.

Spatial and temporal patterns in the modeled average diarrhea incidence were consistent with the observed patterns (Supplementary Figures S3, S4). Figure 2 shows the posterior median estimates of the exponentiated spatial random effect parameters (ϕ_*k*_), resulting in a RR interpretation. These parameters represent excess spatial risk of diarrhea after adjustment for the included covariates (i.e., residual risk). The RRs vary between 0.07 and 6.66. More than a third (38%) of the districts had a relative risk >1; most of the higher risk districts were located in the western part of the country. A south to north gradient increase in RR was also evident when the model was fitted separately for the four agro-ecological zones (Supplementary Figure S5). The lowest RR was present in urban districts within Accra and Kumasi, the two major cities in the country.

## Discussion

Our results highlight the extent to which the spatiotemporal distribution of diarrhea incidence in Ghana is associated with meteorological and socio-demographic factors. All of the explanatory variables we explored exhibited significant associations with diarrhea incidence. However, there were clear differences in the strength and direction of associations between these explanatory variables and diarrhea across the four agro-ecological zones. We find that socio-demographic factors (particularly population density and Gini index) rather than meteorological factors are the strongest predictors of the spatiotemporal distribution of diarrhea risk. The results have identified clusters of districts in Ghana associated with a higher than expected incidence of diarrhea, which could inform policy-makers when planning intervention strategies.

The seemingly high incidence of diarrhea in the northern part of the country (transition and savannah zones) may be a result of multiple factors. One possible explanation is the differential performance of rotavirus vaccine in Ghana, where vaccine impact has been lower in the northern part of the country (40). Rotavirus is the leading diarrheal pathogen among children under 5 years of age, and children account for a high proportion of diarrhea incidence in Ghana (41). Secondly, the low coverage of WASH infrastructure mostly in the transition and savannah zones could be a contributing factor. For instance, the proportion of households without toilets (42) and that practice open defecation (43), which are important risk factors of diarrhea, are high in the transition and savannah zones compared to the rest of the country, particularly in the upper East region, where we observed clusters of diarrhea risk (43). In addition, prevalence of childhood malnutrition is higher in the savannah zone, accounting for 55% of the country's malnutrition (44). The overall high level of poverty and inequality (Gini index) and low level of development (DLT score) across the districts within these two zones (36, 45) also contributed to the estimated high diarrhea risk.

The overall low relative risk of diarrhea in the coastal agro-ecological zone may be attributed to the combination of Gini index, overall level of poverty, population density, and DLT score. Similar to other low-income settings, there is a negative association between poverty and income inequality in Ghana (46, 47). There is a sharp decrease in Gini index and increase in poverty as you move from urban toward peri-urban and rural districts in Ghana. While population density is high in the coastal zone, there is also more highly developed water and sanitation infrastructure compared to other zones. The DLT score is highest in Greater Accra region followed by Central region (both within the coastal zone) (36).

The direction of associations between Gini index and DLT score covariates with diarrhea were significant and similar with the exception of the transition zone. Both covariates can indicate levels of inequality across the districts leading to hotspots of diarrhea. For instance, when Gini index is high, the uneven distribution of wealth can lead to disparity in the level of development (DLT score) particularly related to household WASH infrastructure. Thus, policies aiming at reducing Gini index and increasing DLT score indicators across the districts are likely to reduce the overall diarrhea burden in the country.

Our results indicate a consistent significant negative association between population density and diarrhea incidence in Ghana and across the agro-ecological zones. This finding agrees with a negative association found between cholera risk and population density (48). However, the strength of the association decreases substantially toward the northern part of the country, with about a 2.5-fold difference in the relative risk of diarrhea between the coastal and savannah agro-ecological zones. This difference in the relative risk between these two zones can be partly explained by the relationship between disparity in the level of development (based on DLT score), level of sanitation infrastructure development, and the population growth rate. There is a clear substantial south-north decreasing trend in DLT score (36). The coastal zone is densely populated (including Accra, the capital city), but has more developed water and sanitation infrastructure and a higher DLT score, thus associating with an overall lower risk of diarrhea. On the contrary, the savannah zone exhibits low population density, inadequate WASH infrastructure, and lower DLT scores, associating with a high diarrhea risk. The DLT score could be useful to identify and inform the development of effective interventions to reduce diarrhea in the high-risk districts.

Generally, the relative risk of diarrhea tends to be greater in peri-urban districts compared with urban and rural districts, which agrees with previous studies (49, 50). Population spill-over from the urban districts and high levels of rural-urban migration have led to high population density in peri-urban districts. Peri-urban districts are usually characterized by inadequate sanitation facilities and a high proportion of households living in slum conditions. These slums tend to be hotspots for diarrhea. Similar high diarrhea risk in slums compared with rural settings has been found elsewhere (51, 52). Thus, improvements in sanitation in peri-urban districts in Ghana will likely reduce the diarrhea burden.

We found significant positive associations between surface water presence (a proxy for persistent flooding) and diarrhea for the entire country and across the four agro-ecological zones. This consistent positive association based on a surface hydrology model is different from studies that have reported both positive and negative associations between diarrhea and rainfall/flooding (8, 31–33, 53). Different rainfall regimes can have varied associations with diarrhea depending on the surface antecedent conditions (54). Non-linear associations between rainfall and diarrhea may also be due to differences in climatic conditions and dominant diarrheal pathogens across study areas. Our results highlight that a rainfall-driven surface hydrology model can better account for pathogen concentration and dilution effects, since it incorporates the surface antecedent conditions, thus making it a better predictor of diarrhea risk than rainfall alone. Despite the estimated positive associations across different climatic conditions found in Ghana, future studies should explore whether surface water presence is an important diarrhea risk factor across different climatic conditions and under future climate change.

While the effect of mean temperature on diarrhea has been extensively studied, both significant positive and negative associations with diarrhea risk have been reported (9, 55–58). There are multiple factors that may contribute to this, including climatic conditions of the study region, dominant diarrheal pathogens, demographics of the study population (i.e., all ages, or restricted categories of age groups, particularly children <5 years old) and the temporal resolution of the data. In Afghanistan, positive and negative associations with diarrhea were found with daily and annual temperatures, respectively (9). Despite the overall significant negative association between mean temperature and diarrhea, three (coastal, forest and transition) out of four agro-ecological zones exhibited a positive association, which may be due to the marked difference in climatic conditions between the savannah (which has a prolonged dry season with high temperatures) and the three other zones, as well as the predominant pathogens in the different regions. The impact of temperature on the survival and replication of diarrheal pathogens differs. For instance, viral pathogen activity tends to be highest during the cooler dry season, while bacterial pathogens tend to predominate during the warmer rainy season (25). The similarity in both direction and strength of association for the overall and the savannah zone likely reflects the high diarrhea incidence in this zone.

The diurnal temperature range showed a consistent significant negative association with diarrhea, suggesting that highly variable monthly temperature fluctuations could affect diarrhea incidence in Ghana. A possible reason is that diarrhea peaks during the rainy season, which coincides with the period of low temperature variation. This result differs from the reported positive association between diurnal temperature range and childhood diarrhea in Brisbane, Australia (59), which may be partly due to the study population and dominant diarrheal pathogens. Since diurnal temperature range is an important indicator of climate change, additional studies are required to quantify the impact of diurnal temperature variation across different diarrheal pathogens and age groups under future climates.

There are several factors that could potentially impact our results. First, disparities in access and proximity to health facilities across the districts may affect diarrhea reporting. While those living in districts with readily available health facilities may seek care for both mild and severe cases of diarrhea, those living in districts with limited health facilitates may not seek care except for severe cases due to the distance. There is also the possibility that some people attend hospitals in a different district from where they live. Second, although we observed a significant association between Gini index and diarrhea risk, it is noteworthy to point out that the Gini index was calculated based on 2010 census data; thus, not accounting for temporal changes in Gini index may impact the strength and direction of the associations we found. Third, our data were not stratified by age, and it is possible that associations may differ across age groups. Children under 5 years of age are the most susceptible to diarrhea. Further efforts should be directed at examining how heterogeneity in this age group across the districts may influence the direction of associations between meteorological and socio-demographic factors and diarrhea.

In summary, the burden of diarrhea in Ghana is concentrated in the western part of the country, particularly within the transition and savannah agro-ecological zones. As most of the districts within these two zones exhibit poorer levels of development (based on DLT score), future studies should consider evaluating separately the impact of the indicators (particularly potable water and sanitation coverage) used to estimate the DLT score on household diarrhea incidence. Ghana health sector policy-makers can use the DLT score to formulate appropriate control strategies aimed at prioritizing resource allocation for diarrhea control.

## Data Availability Statement

The diarrhea data can be obtained by contacting the Center for Health Information and Management (CHIM) of the Ghana Health Services. Requests to access these datasets should be directed to Ghana Health Service, info@moh.gov.gh.

## Author Contributions

EA conceived and designed the study, performed data analysis, interpretation of findings, and wrote the paper. JW performed data analysis, interpretation of findings, and revised the manuscript. VP performed data analysis, interpretation of findings, and revised the manuscript. All authors reviewed the manuscript and approved the final version.

## Funding

Research reported in this publication was supported by funding from the National Institutes of Health/National Institute of Allergy and Infectious Diseases (R01AI112970 to VP). The funders had no role in study design, data collection and analysis, decision to publish, or preparation of the manuscript. The content is solely the responsibility of the authors and does not necessarily represent the official views of the National Institutes of Health.

## Conflict of Interest

VP is a member of the WHO Immunization and Vaccine-related Implementation Research Advisory Committee (IVIR-AC) and has received travel reimbursements from Merck and Pfizer for participation in Scientific Input Engagements unrelated to the topic of this paper. The remaining authors declare that the research was conducted in the absence of any commercial or financial relationships that could be construed as a potential conflict of interest.

## Publisher's Note

All claims expressed in this article are solely those of the authors and do not necessarily represent those of their affiliated organizations, or those of the publisher, the editors and the reviewers. Any product that may be evaluated in this article, or claim that may be made by its manufacturer, is not guaranteed or endorsed by the publisher.
