# Supplementary material for: Spatiotemporal Patterns of Diarrhea Incidence in Ghana and the Impact of Meteorological and Socio-Demographic Factors
Source: Front Epidemiol. 2022 Apr 8;2:871232. doi: 10.3389/fepid.2022.871232 (PMC9272077; doi:10.3389/fepid.2022.871232)
Supplement: Supplementary file 1 [file Data_Sheet_1.pdf]

Supplementary Material

Table S1: Summary statistics of raw diarrhea incidence and potential explanatory variables for the four agro-ecological zones.

		**coastal**	
**Variable**	**Mean (SD)**	**Range**	**Median (Quartile)**
Diarrhea (per 10000)	462.0 (339.4)	12.0, 3255.0	370.5 (229.0, 623.0)
dtr (ºC)	0.370 (0.166)	0.002, 0.983	0.341 (0.262, 0.462)
Tmean (ºC)	26.84 (1.268)	23.61, 29.69	27.02 (25.86, 27.85)
wpre	0.006 (0.004)	0.00, 0.019	0.005 (0.003, 0.009)
Gini (%)	36.95 (2.824)	32.10, 46.00	36.60 (34.80, 38.50)
DLT (%)	57.19 (10.86)	15.20, 91.10	58.13 (52.51, 64.98)
Population density (people/km^2)	1552.79 (2735.16)	54.41, 14118.80	419.02 (263.81, 1293.10)

		**Forest**	
**Variable**	**Mean (SD)**	**Range**	**Median (Quartile)**
Diarrhea (per 10000)	589.3 (359.5)	16.0, 2648.0	506.5 (344.0, 756.0)
dtr (ºC)	0.451 (0.229)	0.000,1.270	0.442 (0.283, 0.598)
Tmean (ºC)	26.47 (1.287)	23.02, 30.87	26.45 (25.48, 27.38)
wpre	0.007 (0.004)	0.00, 0.023	0.007 (0.004, 0.010)
Gini (%)	36.22 (4.093)	27.20, 48.10	35.75 (33.30, 38.60)
DLT (%)	57.77 (11.42)	0.00, 100.00	59.19 (53.12, 65.40)
Population density (people/km^2)	481.18 (1634.66)	43.12, 12888.00	142.37 (91.98, 246.78)

		**Transition**	
**Variable**	**Mean (SD)**	**Range**	**Median (Quartile)**
Diarrhea (per 10000)	745.4 (589.0)	4.0, 7304.0	597.0 (418.0, 903.2)
dtr (ºC)	0.541 (0.294)	0.000,1.443	0.537 (0.317, 0.742)
Tmean (ºC)	27.02 (1.53)	23.50, 32.19	26.87 (25.85, 28.07)
wpre	0.006 (0.004)	0.000, 0.021	0.006 (0.002, 0.009)
Gini (%)	41.6 (6.386)	32.0, 64.0	41.1 (37.0, 45.0)
DLT (%)	58.97 (10.87)	21.30, 98.50	60.52 (53.35, 66.23)
Population density (people/km^2)	118.91 (96.32)	15.96, 616.59	90.18 (58.80, 134.91)

		**Savannah**	
**Variable**	**Mean (SD)**	**Range**	**Median (Quartile)**
Diarrhea (per 10000)	768.1 (439.8)	58.0, 3464.0	673.0 (457.0, 971.0)
dtr (ºC)	0.826 (0.290)	0.071, 1.900	0.818 (0.618, 1.022)
Tmean (ºC)	28.73 (2.065)	24.83, 34.30	28.40 (26.98, 30.22)
wpre	0.005 (0.005)	0.000, 0.022	0.004 (0.000, 0.010)
Gini (%)	42.09 (7.535)	30.00, 57.90	42.00 (36.10, 47.50)
DLT (%)	55.04 (11.856)	14.56, 94.70	57.01 (48.48, 63.38)
Population density (people/km^2)	101.66 (135.27)	8.71, 984.86	64.19 (32.49, 114.11)

wpre: surface water presence, dtr: diurnal temperature range, Tmean: mean temperature, DLT score: District League Table score.

Table S2: The Pearson correlation matrix between the covariates for both the complete dataset and across the four agro-ecological zones.

	Complete dataset					
	**Tmean**	**dtr**	**wpre**	**Population density**	**DLT score**	**Gini index**
Tmean	1					
dtr	0.624	1				
wpre	-0.462	-0.388	1			
Population density	-0.081	-0.195	-0.032	1		
DLT score	-0.068	-0.105	0.007	0.053	1	
Gini index	0.23	0.293	-0.063	-0.159	0.117	1

	Coastal					
	**Tmean**	**dtr**	**wpre**	**Population density**	**DLT score**	**Gini index**
Tmean	1					
dtr	0.492	1				
wpre	-0.153	-0.15	1			
Population density	-0.05	-0.26	-0.109	1		
DLT score	0.007	-0.122	-0.012	0.23	1	
Gini index	0.077	0.11	0.02	-0.381	-0.006	1

	Forest					
	**Tmean**	**dtr**	**wpre**	**Population density**	**DLT score**	**Gini index**
Tmean	1					
dtr	0.52	1				
wpre	-0.382	-0.259	1			
Population density	-0.004	-0.076	-0.028	1		
DLT score	-0.019	-0.051	0.025	-0.059	1	
Gini index	0.111	0.131	0.019	-0.079	0.017	1

	Transition					
	**Tmean**	**dtr**	**wpre**	**Population density**	**DLT score**	**Gini index**
Tmean	1					
dtr	0.672	1				
wpre	-0.51	-0.394	1			
Population density	-0.241	-0.426	0.024	1		
DLT score	-0.131	-0.202	-0.016	0.145	1	
Gini index	0.029	0.136	-0.017	-0.142	0.077	1

	Savannah					
	**Tmean**	**dtr**	**wpre**	**Population density**	**DLT score**	**Gini index**
Tmean	1					
dtr	0.396	1				
wpre	-0.593	-0.611	1			
Population density	0.051	-0.07	0.005	1		
DLT score	0.031	0.04	-0.031	0.125	1	
Gini index	0.074	0.116	-0.023	0.216	0.365	1

wpre: surface water presence, dtr: diurnal temperature range, Tmean: mean temperature, DLT score: District League Table score.

Table S3. The Geweke diagnostic test.

Variable	Gweke diag
Intercept	-0.3
wpre	-0.5
dtr	-0.1
Tmean	-1.7
Population density	-0.3
Gini index	0.9
DLT score	-0.8

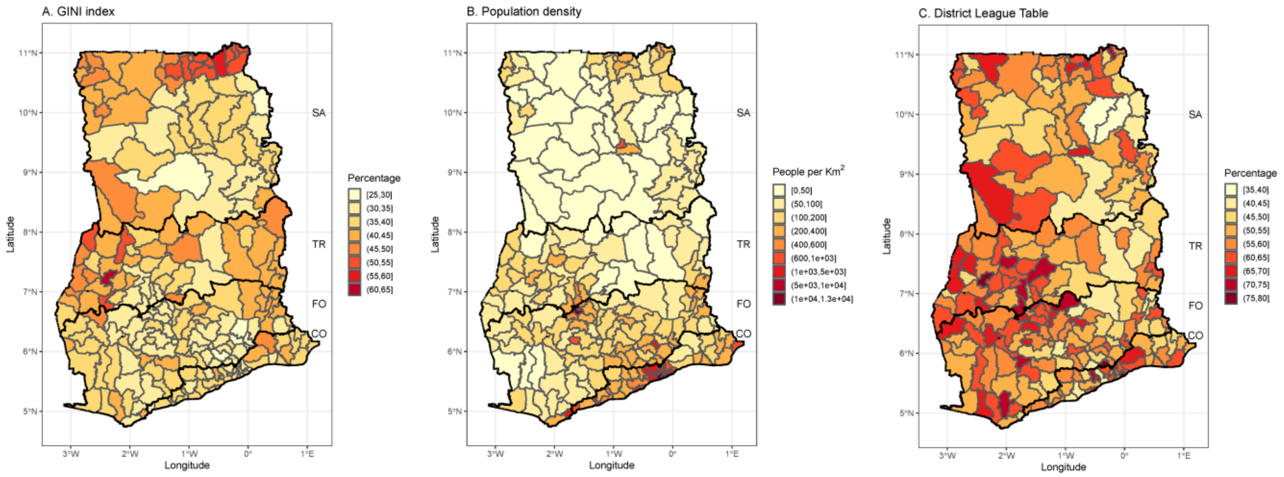

Figure S1. Spatial patterns of the socio-demographic explanatory variables used in the model. **(A)** Gini index, **(B)** population density, and **(C)** District league Table (DLT) score. The black bold line indicates agro-ecological zone boundaries. Abbreviations: CO, Coastal zone; FO, Forest zone; TR, Transition zone; SA, Savannah zone.

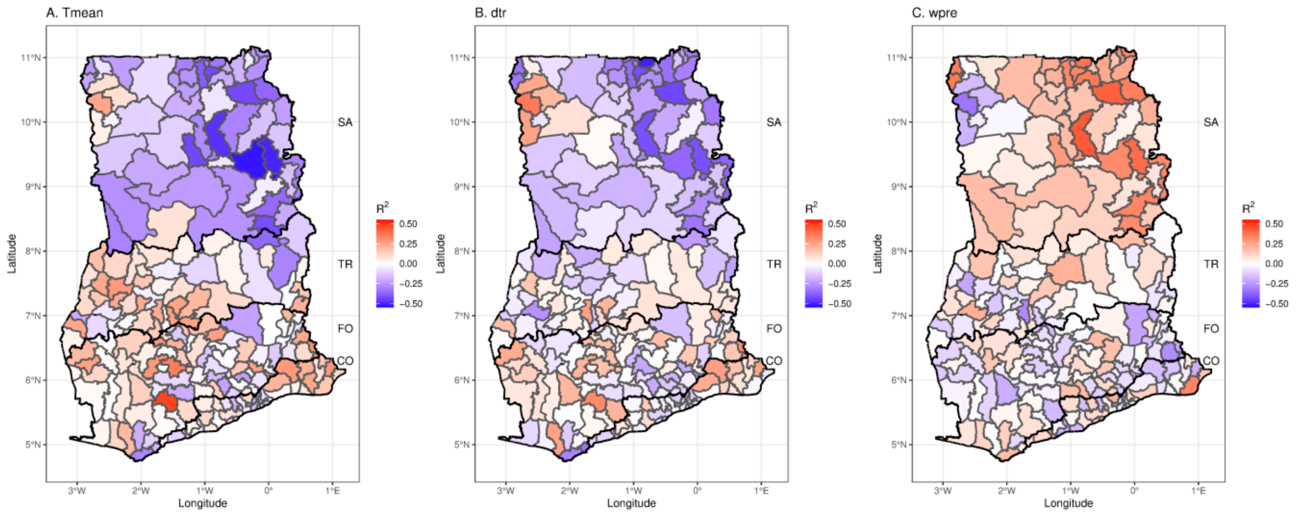

Figure S2. Spatial maps of correlation coefficients between monthly diarrhea incidence and the meteorological factors. **(A)** Mean temperature (Tmean), **(B)** diurnal temperature range (dtr), and **(C)** surface water presence (wpre). The black bold line indicates agro-ecological zone boundaries. Abbreviations: CO, Coastal zone; FO, Forest zone; TR, Transition zone; SA, Savannah zone.

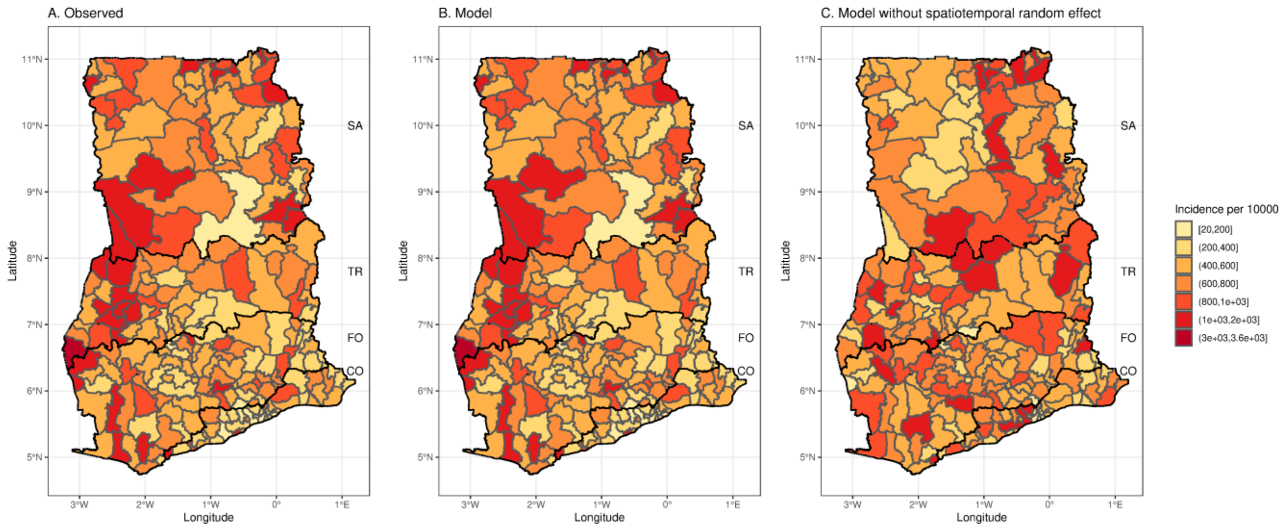

Figure S3: Spatial map of average diarrhea incidence over 2012-2018. **(A)** Observed incidence and **(B)** model-predicted incidence per 10,000 people per year. **(C)** Model-predicted incidence without spatiotemporal autocorrelated random effects. The black bold line indicates agro-ecological zone boundaries. Abbreviations: CO, Coastal zone; FO, Forest zone; TR, Transition zone; SA, Savannah zone.

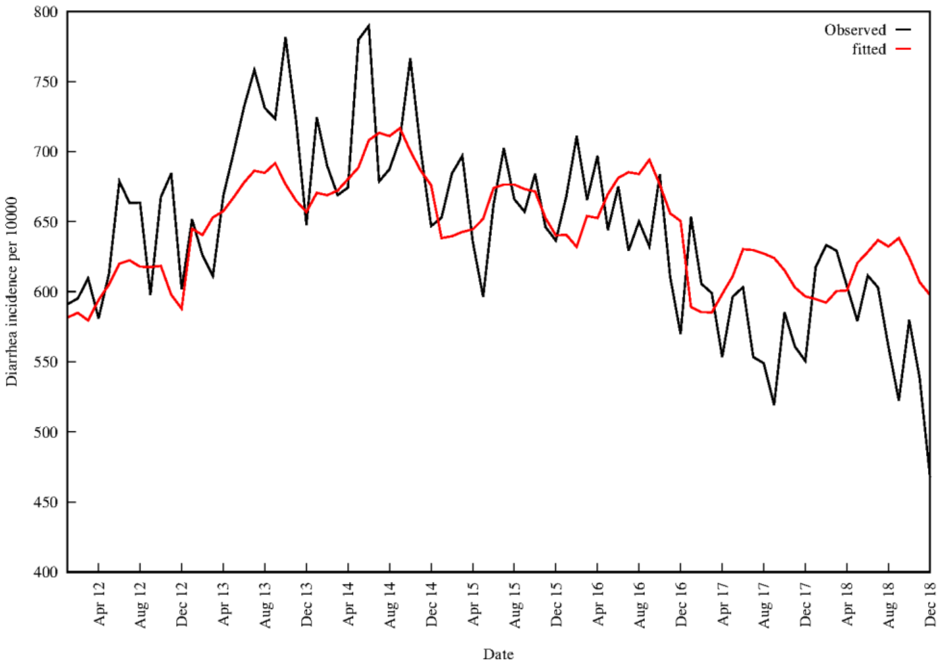

Figure S4: Comparison of observed and model predicted temporal patterns of average diarrhea incidence over the study period.

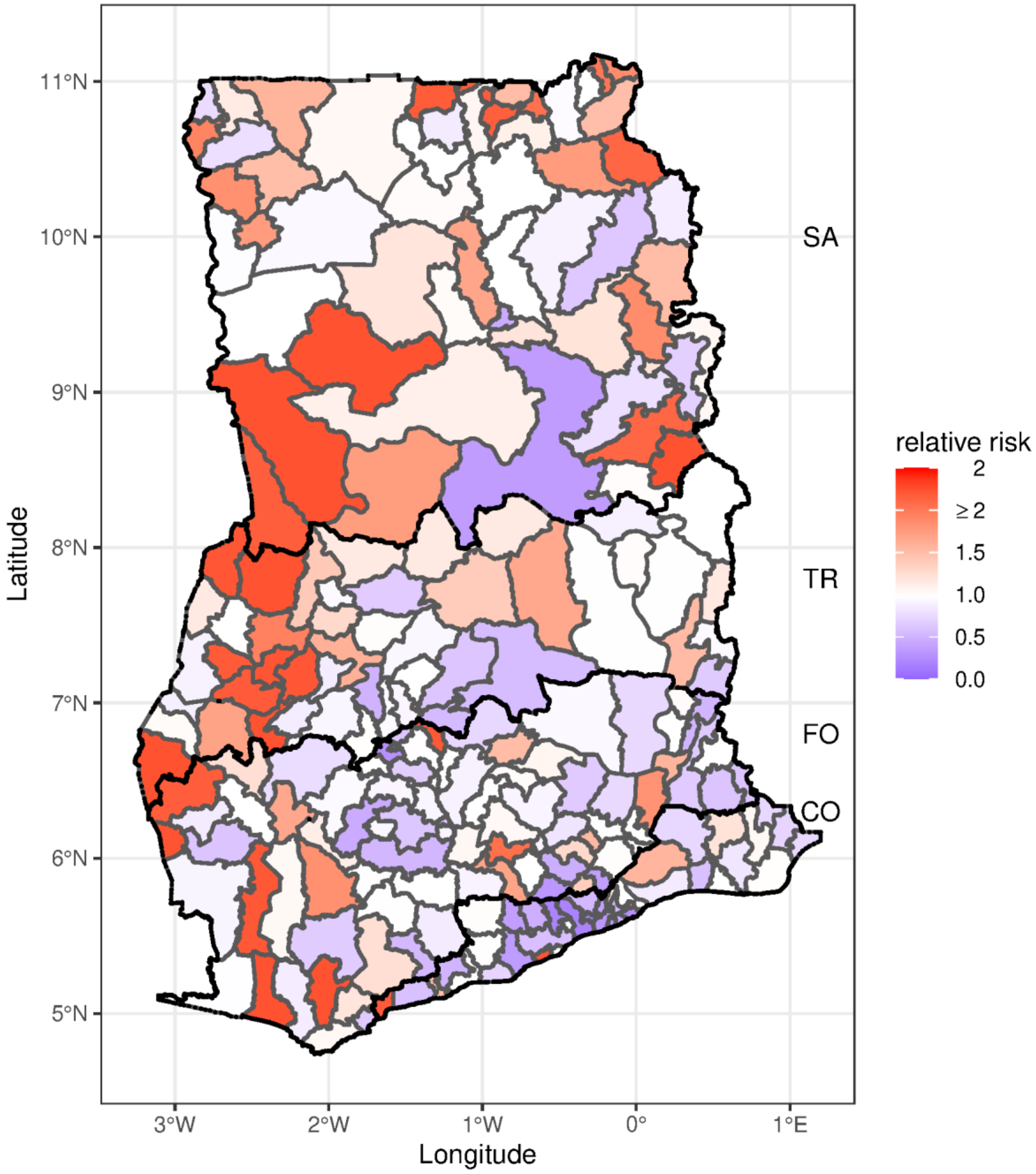

Figure S5: Map of posterior median estimates of the spatial random effects across the four agro-ecological zones. Districts with relative risk <1 are in blue and those with relative risk >1 are in red. The black bold line indicates agro-ecological zone boundaries. Abbreviations: CO, Coastal zone; FO, Forest zone; TR, Transition zone; SA, Savannah zone.
